# Assessment of cardiopulmonary resuscitation equipment in resuscitation trolleys in district hospitals in Botswana: A cross-sectional study

**DOI:** 10.4102/phcfm.v11i1.2029

**Published:** 2019-10-17

**Authors:** Billy M. Tsima, Lakshmi Rajeswaran, Megan Cox

**Affiliations:** 1 Department of Family Medicine and Public Health, University of Botswana, Gaborone, Botswana; 2 School of Nursing, University of Rwanda, Kigali, Rwanda; 3 Faculty of Medicine and Public Health, Sydney University, New South Wales, Australia

**Keywords:** cardiac arrest, resuscitation trolley, resuscitation, district hospitals, Botswana

## Abstract

**Introduction:**

Successful cardiopulmonary resuscitation (CPR) relies, in part, on the availability and the correct functioning of resuscitation equipment. These data are often lacking in resource-constrained African settings.

**Aim:**

To assess the availability and the functional status of CPR equipment in resuscitation trolleys at district hospitals in Botswana.

**Setting:**

The study was conducted across four district hospitals in Botswana.

**Methods:**

A cross-sectional study was conducted using a checklist adopted following the Emergency Medical Services of South Africa (EMSSA) guidelines, modified and contextualised to Botswana.

**Results:**

All the four district hospitals had inadequate number of CPR equipment available in the resuscitation trolleys. The overall availability of drugs and equipment ranged from 19% to 31.1%. Availability of equipment needed for maintaining circulation and fluids ranged from 27% to 49%, while availability of items for airway and breathing ranged from 9.2% to 24.1%. The overall availability of essential drugs for resuscitation was only 20.4%, and in some wards expired drugs were kept in the trolley. Out of 40 wards that participated in the study, only 10 kept CPR algorithms in the resuscitation trolley. The resuscitation trolley was checked on a daily basis only in the critical care units.

**Conclusion:**

The resuscitation trolleys were not maintained as per standards. Failure to improve the existing situation could negatively impact the outcome of CPR. Evidence-based standard checklists for resuscitation trolleys need to be enforced to improve the quality of CPR provision in district hospitals in Botswana.

## Introduction

The precise timing of cardiopulmonary arrest is generally unpredictable even though the arrest may follow a predictable failure to treat known physiological abnormalities.^1^ Successful cardiopulmonary resuscitation (CPR) relies, in part, on the availability and correct functioning of the resuscitation equipment.^2^ To this effect, resuscitation trolleys were conveniently developed to store key resuscitation equipment, and to facilitate wheeling of the equipment to an emergency when required.^3^ The National Patient Safety Agency (NPSA) has identified issues with missing or non-functional resuscitation equipment in resuscitation trolleys as deficiencies compromising patient safety and leading to death among in-patients requiring CPR.^4^

Several international studies have confirmed that effective and safe CPR is reliant on the immediate availability of resuscitation equipment.^5,6,7^ Guidelines published by several resuscitation councils include advice on airway management, chest compressions, defibrillation and many aspects of CPR. These guidelines also refer to items constituting the equipment required for successful CPR and have been used by resuscitation teams in hospitals to improve in-patient provision of CPR. Evidence from resourced settings suggests that a wide variation exists in survival after CPR, with the best outcomes achieved in specialised units such as the accident and emergency (A&E) department and coronary care unit.^8,9^ Although the rational use of drugs and defibrillation in resuscitation has long been standardised according to international guidelines based on resuscitation science evidence, standardisation of resuscitation equipment has often lagged behind.^9^

Deficiencies in the availability and the maintenance of resuscitation equipment have been widely reported in the literature.^7,9^ Studies conducted in various African countries (Botswana,^10^ Ghana,^11^ Kenya,^12^ Namibia^13^ and South Africa^14^) as well as Asia (Thailand,^15^ India^16^), the United Kingdom^17^ and the United States^18^ indicate that the non-availability of equipment and drugs in the resuscitation trolley causes delay in initiating CPR.

The Disease Control Priorities in Developing Countries Project estimates that 45% of deaths and 36% of Disability-Adjusted Life Years (DALYs) in low- and middle-income countries could be addressed by the implementation of emergency care systems.^19^ The highest burden of diseases in developing countries often exists at the community level, especially in primary care settings with inadequate resuscitation resources or lack of timely transport services to secondary and tertiary hospitals.^20^ Adequate availability of resuscitation equipment, drugs and well-prepared health care professionals able to provide timely care are important to maintain the chain of survival for patients requiring emergency medical care. In Botswana, standardised checklists for resuscitation trolley contents have only recently been adopted and used at a single referral hospital. The extent to which district hospitals in Botswana measure up to the checklist is unknown. Efforts to improve CPR outcomes require information about the availability and functionality of resuscitation equipment reserved for CPR and historically stored in resuscitation trolleys. We therefore aimed to assess the availability and functional status of CPR equipment forming part of the resuscitation trolley at district hospitals in a resource-constrained setting.

## Methods

### Research method and design

A cross-sectional study was conducted to audit the contents of resuscitation trolleys in four district hospitals in Botswana.

### Setting

Botswana is a landlocked country with an estimated population of 2.1 million in Southern Africa sharing its borders with South Africa, Namibia, Zimbabwe, Angola and Zambia.^21^

The health care system in Botswana is modelled on the primary health care (PHC) philosophy and adopts many initiatives and guidelines of the World Health Organization (WHO). Botswana has five levels of health care of increasing complexity from a health post, primary care clinic, primary hospital, district hospital to referral hospital. There are three referral hospitals, seven district hospitals, 16 primary hospitals, two mission hospitals, two private hospitals and two mine hospitals located in different parts of Botswana.

District hospitals in Botswana play an important role in supporting primary health care and are established in major towns and have a bed capacity of 250–300. The district hospitals serve populations between 50 000 and 70 000. Preventive, promotive, curative, rehabilitative services, as well as 24-h emergency services and advanced trauma and cardiac life support are provided within the district hospital system.

In district hospital settings in Botswana, a centralised response system does not exist, and CPR is provided by clinicians working in the clinical area where cardiopulmonary arrests occur. The resuscitation trolley in the ward is thus the primary source of CPR equipment needed to respond to cardiac arrests in the ward.

Among the seven district hospitals in Botswana, four were conveniently chosen for this study. All the four district hospitals are in different geographical locations along the national highway linking the northern and southern parts of the country. The district hospitals were chosen for the current study, as they had been recently upgraded with modern facilities such as intensive care units (ICUs) and A&E departments to expand services and decongest the two main referral hospitals in Botswana. Furthermore, the four hospitals were engaged in accreditation exercises through the Council for Health Services Accreditation of South Africa (COHSASA). Maintenance and availability of equipment in resuscitation trolleys is one of the accreditation requirements in these quality improvement exercises.

### Sample size and sampling

The sample size was exhaustive and included 40 wards across the four district hospitals. These include A&E departments, ICUs, male and female medical wards, male and female surgical wards, male and female orthopaedic wards, paediatric wards, and obstetrics and gynaecology wards. Convenience sampling technique was used to audit the resuscitation trolleys.

### Data collection instrument and procedure

A checklist adopted by one of the local referral hospitals following the EMSSA 2010 guidelines, which had been modified and contextualised to Botswana, was used to audit the resuscitation trolleys for all district hospitals in the study.^22^ Equipment was scored as being present (1) or absent (0) and a percentage of the recommended quantity was calculated for each group. Equipment that was available but not working was scored as being absent.

Each facility was contacted a week prior to the scheduled data collection visit. The purpose of the study was explained to the ward managers. Two researchers (a critical care nurse and an emergency medicine physician) visited the four district hospitals and audited the equipment, drugs and the resuscitation trolleys. Each visit took about 3–5 h to check all the resuscitation trolleys in each hospital against the checklist.

### Reliability and validity

Two critical care nurses, an emergency medicine physician and an anaesthetist critically reviewed the checklist. Consensus about the contents of the checklist was reached and all agreed that the listed items were essential for inclusion in resuscitation trolleys in Botswana.

The checklist was pre-tested in two wards that did not participate in the study. No problems were identified during the pre-testing, and the checklist was consequently endorsed.

### Data analysis

Data were analysed using Statistical Package for Social Sciences (version-22). Microsoft excel was used to generate figures and graphs. Figures and tables display the data expressed in percentages for comparison purposes.

### Ethical consideration

Permission to conduct the study was granted by the four district hospitals, the research unit in the Ministry of Health, Botswana, and the Office of Research and Development, University of Botswana (UBR/RES 3/2). No person other than the investigators was involved in the audits of the resuscitation trolleys in the selected wards of the four hospitals. Data were kept in a protected environment and only the principal investigator had access to the information.

## Results

### Characteristics of the hospitals

All the hospitals had A&E departments, and medical, surgical, labour and post-natal wards. Only two hospitals had an ICU and one hospital had a high care unit. A total of 40 wards were assessed across the four district hospitals.

### Overall availability of essential equipment at the four district hospitals

The checklist prescribes for a total of 80 different categories of emergency equipment for each resuscitation trolley and a total of 510 pieces of equipment, including different sizes of items in each category of equipment in the resuscitation trolley.

**FIGURE 1:**
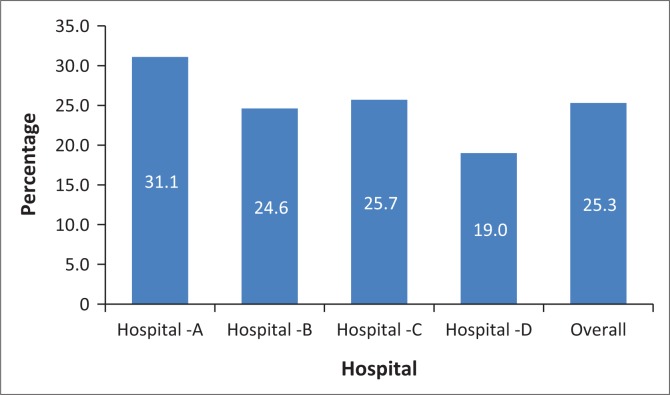
Overall distribution of equipment and drugs.

The overall distribution of the equipment, drugs and miscellaneous items ranged from 19% to 31.1%. Hospital A had the highest proportion (31.1%) compared to the other three hospitals (B: 24.6%, C: 25.7%, D: 19.0%). However, this was still below the threshold recommended in the standard checklist (Figure 1).

The distribution of CPR equipment available in resuscitation trolleys stratified by clinical area ranged from 60% to 25%. The higher proportions were recorded for the ICUs (60%) and A&E departments (55%) compared to the other wards.

Availability of equipment in the resuscitation trolleys in the medical and surgical wards at all the four hospitals ranged from 25% to 30%. In some wards, bag valve masks, recommended sizes of endotracheal tubes, introducers for intubation, Magill forceps and portable suction machines were not found in the trolley, and portable oxygen cylinders were not seen close to the nurses’ station.

### Distribution of the equipment for circulation and fluid resuscitation

**FIGURE 2:**
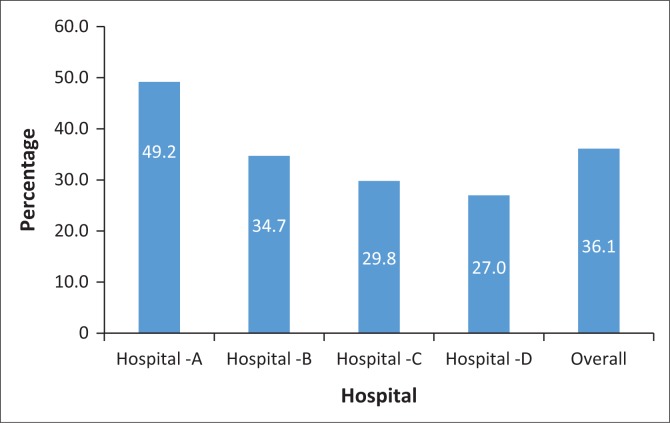
Distribution of equipment needed for circulation and fluid.

The proportions of available equipment for circulation and fluid resuscitation among the four district hospitals are shown in Figure 2. The average proportion available was 36% and ranged from 27% to 49.2%.

Hospital A had the highest availability (49.2%) of the equipment needed for maintaining circulation and fluid, while hospital D (27.0%) had the lowest availability among all the four hospitals. In many of the wards, intravenous fluids (IVs) and sets were found outside the trolley. In some wards in hospital C, makeshift trolleys were noted to be in use and a small tray was substituted for a resuscitation trolley. In more than three wards in each district hospital, automated external defibrillators (AEDs) were not available. Where available, the AED was being shared among wards.

### Airway and breathing

According to the standardised checklist, 20 items of equipment are necessary to maintain the airway and breathing and should be available in the resuscitation trolley.

**FIGURE 3:**
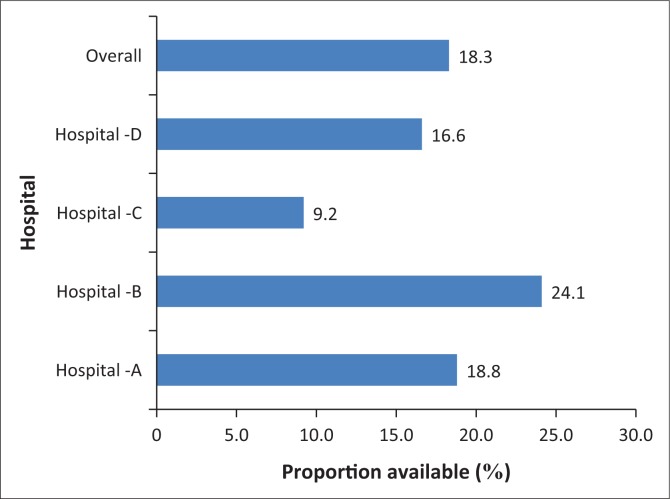
Distribution of equipment needed for airway and breathing.

The proportions of the available equipment for airway and breathing are shown in Figure 3. Hospital B had the highest proportion (24.1%) of specified items for airway and breathing, and hospital C had the least (9.2%) number of items in the resuscitation trolley. Airway adjuncts like bag valve mask, nasopharyngeal airway and elastic gum bougies were not found at all in some wards.

**FIGURE 4:**
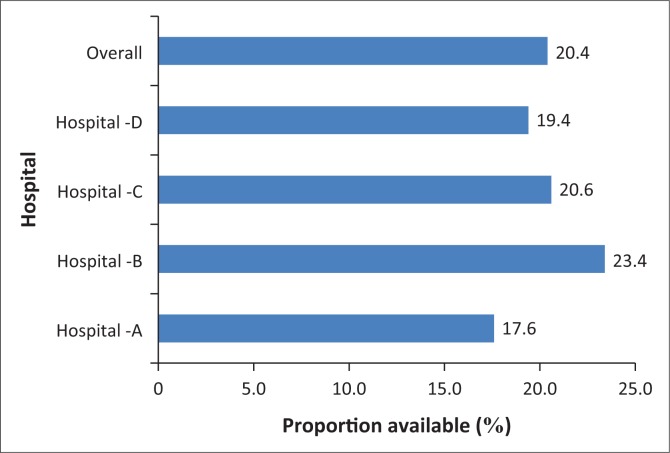
Distribution of emergency drugs.

According to the checklist, 25 drug items should always be available in the resuscitation trolley. A comparison of the availability of the drugs in the four district hospitals is shown in Figure 4. Hospital D had 19.4% and hospital B had 23.4% availability of drugs. Hospital A (17.6%) had the lowest distribution, while hospital C had a slightly better distribution (20.6%) of drugs. The audit also indicated that the availability of drugs was the highest in A&E departments and ICUs (92%). In two of the district hospitals, non-recommended and expired drugs were kept in the trolleys. Overall, only 20.4% of the required drugs were available across all four hospitals. Cardiac drugs such as adenosine and amiodarone were not available in all the four district hospitals.

### Miscellaneous resuscitation items

**FIGURE 5:**
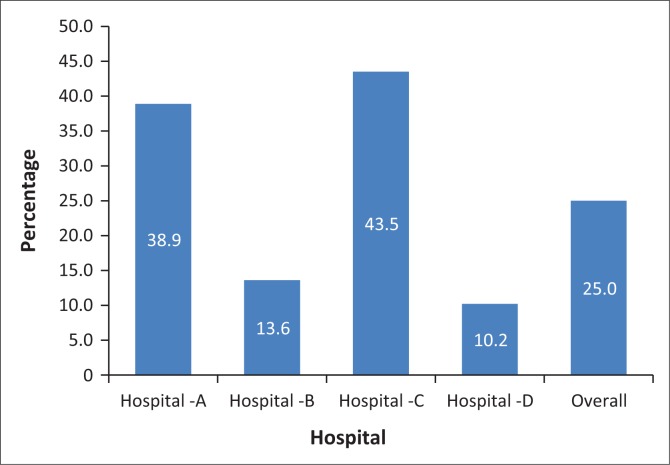
Distribution of miscellaneous items.

As per the standardised guidelines, suction machines, heavy duty scissors, equipment necessary for following universal precautions (Goggles, Gowns), adult and paediatric semi-rigid collars and medication stickers should be available in the resuscitation trolley. Out of the 40 wards participating in the study, only 10 wards kept algorithms in the resuscitation trolley. The cervical collar was available only in one of the four district hospitals’ A&E department. In many wards, the suction machine was shared among the wards. Overall, only 25% of the requisite miscellaneous resuscitation items were available (Figure 5).

### Checking of the resuscitation trolley

The resuscitation trolley checklist booklet from each ward in the four district hospitals indicated that the trolley had been checked on a daily basis only in the A&E department and in the ICU. Thus, only 15% (6/40) of the wards complied with the daily checking recommendation.

## Discussion

Our results highlight an inadequate availability of drugs and CPR equipment in resuscitation trolleys at the four district hospitals in Botswana. The overall availability of drugs and equipment in the four district hospitals ranged from 19% to 31.1%, far below the ideal 100% availability. Similar findings of low proportions of CPR equipment in resuscitation trolleys have been reported in studies conducted in the United Kingdom,^17^ Sri Lanka,^23^ Namibia^13^ and Ghana.^11^ Critical care units had a better distribution of CPR equipment compared to other wards included in our study. However, critical care units were underrepresented in our sample as only two out of 40 wards were designated as ICUs. Other researchers have reported similar findings of higher proportions of resuscitation equipment availability in the clinical areas of critical care and A&E.^9^ A large proportion of cardiac arrests occur in these clinical areas, and thus the availability of CPR equipment may be a reflection of extra vigilance taken by health care workers to replenish resuscitation trolleys in these areas compared to the other clinical areas with less frequency of cardiac arrests.^8^ Although our results indicate a low overall availability of CPR drugs and equipment in the district hospitals in Botswana, a significant proportion of the equipment noted to be absent in the resuscitation trolleys may not be a basic need at district hospitals. Specialised equipment such as McGill’s forceps and bougies for intubation included in the checklist are less of a priority in resuscitation trolleys placed in general wards but essential in operating theatres.

Studies conducted in various African countries like Sierra Leone,^24^ Kenya^12^ and Malawi^25^ showed that equipment necessary for airway management, drugs, consumables such as syringes, IV fluids and cervical collars were irregularly available to provide emergency resuscitation because of stock-outs. Therefore, attention to improve the availability of the essential items should be given priority in low- and middle-income countries, and solutions to prevent depletion of these should be sought.

The finding of reduced and non-availability of the equipment necessary for airway and breathing such as suction machine, bag valve mask and laryngoscope, which are essential components of the resuscitation trolley, raises a question about the adequacy of CPR provision in these settings. A survey conducted in the UK hospitals reported that the availability of equipment for respiratory support was not uniform and varied across hospitals.^26^ Missing and broken items were identified in some of the resuscitation trolleys in the UK hospitals included in that survey. This may be an indication that regular checking and stocking of resuscitation trolleys is not adequately done. The responsibility of checking and replenishing the resuscitation trolley should ideally be given to clinicians trained in CPR such as nurse anaesthetists as they are more familiar with the equipment than junior and non-specialised nurses. Such an intervention is likely to be of low cost but high impact if nurse anaesthetists in district hospitals are scheduled to conduct ward rounds to specifically check resuscitation trolleys as a quality improvement measure.

In our study in district hospitals in Botswana, most of the wards had standardised resuscitation trolleys. However, at one of the district hospitals, the trolleys had been altered, immobilised and placed on top of a table located in the nurses’ station. In some wards, oxygen cylinders were often kept in different locations. Immobile resuscitation trolleys and oxygen cylinders in unfamiliar locations could cause significant delays in resuscitation attempts.

First-line CPR drugs like amiodarone and adenosine were not available in most of the wards. These findings similar to studies conducted in Sri Lanka^23^ and Malawi^25^ suggest that the deficit is not unique to Botswana. The high level of non-availability of resuscitation drugs as well as storage of expired drugs in the trolley is a cause for concern as this can jeopardise the survival of the cardiac arrest patients.

Our findings further reveal that expired drugs and drugs not required during resuscitation were unnecessarily kept in resuscitation trolleys. Because resuscitation of a cardiac arrest victim presents a stressful situation, medication errors are common during CPR.^27^ Medication errors can occur if the resuscitation trolley is not checked regularly for expired drugs. The efficiency of emergency drugs declines beyond the expired date and can render management of emergencies ineffective.^27^ A study conducted in the United States documented that medication errors during cardiac arrest are 39 times more likely to result in harm and 51 times more likely to result in death.^28^ The finding of surplus medication, such as seen in our study, can make locating key items difficult in an emergency situation and has been associated with delays in the resuscitation process.^7^

In our study, most of the wards were missing resuscitation equipment items such as AEDs and batteries. In some wards, the AEDs were either missing or shared between two wards. Similar situations have been reported in Namibia^12,13^ and South Africa.^14^ Adequate number of electrical and hardware spare parts and batteries should be always kept in the trolley because the concept of resuscitation trolleys was developed to maximise efficiency in critical situations.^29^ The AED is expected to be available in all the wards, as it is an essential step in the management of cardio-respiratory arrest.^30^ A delay in defibrillation of more than 2 min in patients whose initial in-patient cardiac arrest rhythm is ventricular fibrillation or pulseless tachycardia has been associated with lower survival from adult in-hospital cardiac arrest.^31^

The infrequent checking of the resuscitation trolleys discovered in the four district hospitals compromises patients’ safety. Our findings indicate that the resuscitation trolleys were checked on a daily basis only in the critical care units of these hospitals (A&E, ICU). The UK Resuscitation Council recommends each ward should be responsible for checking its own resuscitation equipment preferably on a daily basis. Similarly, the EMSSA guidelines recommend daily checking of resuscitation equipment in emergency trolleys. Malfunctioning of the equipment should be reported to the concerned authorities immediately and replenished as needed. Similar findings of infrequent checking of resuscitation trolleys have been reported elsewhere. A study by Smith et al.^17^ and Kudavidanage et al.^23^ identified that some resuscitation trolleys were checked once in 3 days and some remained unchecked even up to 9 days. The reasons for infrequent checking identified in a study conducted in India were work load, decreased accountability and inefficient monitoring.^16^ According to Davies et al., reducing the complexity of the system, optimising information, wise automation, use of constraints and mitigating the unwanted side effects of change would enhance the adherence to checking procedures.^7^ Although daily checking of resuscitation equipment is considered good practice and is in place at many institutions, its importance has been questioned.^7^ Some resuscitation authorities have begun to relax the recommendation for daily checks and are recommending that the frequency of checking be determined locally.^32^

Ideally, all the hospitals and wards should adhere to the latest guidelines developed by professional organisations such as resuscitation councils, and these should be readily available to all health care professionals. In the current study, guidelines were available in only 10 wards of the four hospitals. Resuscitation algorithms or guidelines are crucial as they describe the best recommended practices based on the current scientific evidence^33^ and have been shown to help improve survival rates and the quality of life.^34^

Innovative solutions for maintaining adequate CPR equipment in resuscitation trolleys have included the re-designing of the trolley so as to reduce the time taken to locate the equipment^35^ as well as implementing a sealed tray system used in conjunction with a database that tracks equipment.^7^ Although these innovations lead to sustained improvement in the availability of resuscitation equipment in resuscitation trolleys, their generalisability to resource-constrained settings is limited by their high cost. Research and development efforts should focus on cost-effective solutions for the maintenance of the resuscitation trolley, which would ensure that the availability of CPR equipment and drugs is not a barrier to resuscitation of the cardiac arrest patient.

## Limitations of the study

This study was conducted in four public district hospitals in Botswana. The findings cannot be generalised to other district hospitals in Botswana. However, district hospitals in Botswana are generally comparable as they share the same source of funding and resources. The district hospitals were alerted regarding data collection visits the week prior, so it is plausible that the resuscitation trolleys were prepared in anticipation of our visit possibly introducing a Hawthorne effect in our findings. An unannounced data collection visit could have reduced the risk of this potential bias and reflected a more accurate assessment of resuscitation capability in the district hospitals in Botswana. Because non-functional equipment was taken to be absent, our results may not accurately capture the true availability of equipment on a regular basis as the non-functional status may have been temporary. However, the binarisation of data with non-functional equipment recorded as absent was determined *a priori*, as it is well recognised that correct functioning of equipment is critical to CPR success.^2^

The use of the same checklist for all wards regardless of the level of care offered at the ward may limit robust comparison between different wards. For instance, an ICU offers higher level of care than a general ward and thus is expected to be better equipped to start with. Holding the general ward to the same standards as an ICU may have unnecessarily penalised the former. Our results comparing critical care wards with general wards should therefore be interpreted with caution in light of this limitation.

This study did not collect CPR rates or cardiac arrest outcome rates in any of the four district hospitals. These hospitals are in different parts of the country and may have very different critical care patient admission guidelines. Future studies should collect CPR rates data and compare cardiac arrest outcome rates among district hospitals as well as data regarding who checks the resuscitation trolleys and proportions of clinical staff with training in CPR such as Basic Life Support or Advanced Cardiovascular Life Support in resource-constrained settings.

## Conclusion

In the four participating district hospitals, resuscitation trolleys were not maintained as per international standards. The availability of CPR equipment and drugs was consistently low among the district hospitals evaluated in this study. Failure to improve the existing situation would impact negatively on the outcome of CPR. Evidence-based standard checklists for resuscitation trolleys need to be enforced to improve the quality of CPR provision in district hospitals in resource-constrained settings. Following the results of this analysis from Botswana, quality improvement measures targeting the upkeep of resuscitation trolleys in the different wards of district hospitals and tasking clinicians trained in CPR, such as nurse anaesthetists, for checking the contents and the functionality of the equipment in resuscitation trolleys are recommended.
